# Unplanned hospital readmission after surgical treatment for thoracic spinal stenosis: incidence and causative factors

**DOI:** 10.1186/s12891-021-03975-6

**Published:** 2021-01-20

**Authors:** Hui Wang, Longjie Wang, Zhuoran Sun, Shuai Jiang, Weishi Li

**Affiliations:** 1 Orthopaedic Department of Peking, University Third Hospital, 49 Huayuan North Road, Haidian District, 100191 Beijing, China; 2 Beijing Key Laboratory of Spinal Disease Research , Beijing, China; 3 Engineering Research Center of Bone and Joint Precision Medicine Ministry of Education , Beijing, China

**Keywords:** Unplanned hospital readmission, Thoracic spinal stenosis, CSF leakage, Pseudomeningocele

## Abstract

**Background:**

To assess the incidence and causative factors of unplanned hospital readmission within 90 days after surgical treatment of thoracic spinal stenosis (TSS).

**Methods:**

Hospital administrative database was queried to identify patients who underwent surgical treatment of TSS from July 2010 through December 2017. All unplanned readmissions within 90 days of discharge were reviewed for causes and the rate of unplanned readmissions was calculated. Patients of unplanned readmission were matched 1:3 to a control cohort without readmission.

**Results:**

Twenty-one patients (incidence of 1.7 % in 1239 patients) presented unplanned hospital readmission within a 90-day period and enrolled as the study group, 63 non-readmission patients (a proportion of 1: 3) were randomly selected as the control group. Causes of readmission include pseudomeningocele (8 patients; 38 %), CSF leakage combined with poor incision healing (6 patients; 29 %), wound dehiscence (2 patient; 9 %), surgical site infection (2 patients; 9 %), spinal epidural hematoma (1 patient; 5 %), inadequate original surgical decompression (2 patients; 9 %). Mean duration from re-admission to the first surgery was 39.6 ± 28.2 days, most of the patients readmitted at the first 40 days (66.7 %, 14/21 patients). When compared to the non-readmitted patients, diagnosis of OPLL + OFL, circumferential decompression, dural injury, long hospital stay were more to be seen in readmitted patients.

**Conclusions:**

The incidence of 90-day unplanned readmission after surgical treatment for TSS is 1.7 %, CSF leakage and pseudomeningocele were the most common causes of readmission, the peak period of readmission occurred from 10 to 40 days after surgery, patients should be closely followed up within this period.

## Background

Unplanned hospital readmission following surgical procedures are undesirable outcomes for patients and medical providers, represent a heavy financial burden upon healthcare systems, and have been a target for improving care and reducing costs [1–3]. Readmission rates and causes have been reported for a number of spinal surgical procedures. For cervical spine, Akamnonu et al. reported 90-day readmission rates of 1.5 % and 3.13 % in 640 and 128 patients undergoing anterior and posterior degenerative cervical spine procedures [4]. For lumbar spine, Chibuikem et al. reported a readmission rate of 2.82 % in a cohort of 140 Medicare patients who underwent surgery for degenerative lumbar stenosis within the 90-day postdischarge period [5]. Surgical site infections (SSI), wound disruptions, medical comorbidity are cited as common causes of hospital readmission [6–8]. A better understanding of the causative factors for readmission may help improve patient counseling and identify high-risk subjects that could be targeted with strategies to prevent readmission [9, 10].

Myelopathy caused by thoracic spinal stenosis (TSS) is much less common than in the cervical and lumbar spine, since the thoracic spine is relatively stable [11]. From an epidemiologic standpoint, there are populations in which this condition is more prevalent, such as East Asians and particularly Chinese, Japanese, and Korean populations [12, 13]. There are also case reports in Western populations, but the number of cases is limited [14, 15]. As the thoracic spinal canal is relatively narrow and the thoracic cord has a poor blood supply, severe neurological symptoms may develop if TSS is not treated promptly [14]. Once the thoracic myelopathy is symptomatic, it is usually progressive and refractory to conservative treatment. Due to the unique anatomical characteristics of the thoracic spine, the incidence of postoperative complications could not be underestimated, such as acute neurologic deterioration, cerebrospinal fluid (CSF) leakage, hematoma, infection [16–19]. Whether and which of these complications may increase the rate of unplanned readmission followed thoracic decompression surgery is unknown, because there has been no study on the rate of unplanned readmission after surgical treatment for TSS due to the low prevalence of the condition.

The purpose of this study is to demonstrate the incidence and causative factors of unplanned hospital readmission after surgical treatment of TSS within 90 days.

## Methods

### Patient

After receiving approval from our institutional review board, we reviewed the clinical records of all patients who underwent surgical treatment of TSS from July 2010 through December 2017 at our institution. Inclusion criteria: (1) primary diagnosis of TSS. (2) preoperative thoracic CT and MRI were available. (3) minimum follow up of two years. Exclusion criteria: (1) thoracic spinal fracture, tumor, infectious disease, scoliosis or kyphosis. (2) first thoracic spine surgery performed at other hospitals. 1239 patients who met both the inclusion and exclusion criteria were retrospectively reviewed. Unplanned hospital readmission was defined by any hospital readmission for any reason within 90 days of initial surgery. Patients presented unplanned hospital readmission were included as the study group, causative factors of the unplanned readmission were recorded. Patients of unplanned readmission were matched 1:3 to a control cohort without readmission.

### Data collection

The following data was collected in all patients of the two groups: patient characteristics including age at first surgery, sex, body mass index (BMI), diagnosis, number of levels involved, comorbidity (hypertension, diabetes, cardiovascular disease). Surgical characteristics include surgical method, surgery time, estimated blood loss, extubation time, CSF leakage, length of hospital stay.

Patient diagnosis was divided into the following categories according to the radiological findings: thoracic disc herniation (TDH), ossification of posterior longitudinal ligament of thoracic spine (OPLL), ossification of thoracic ligamentum flavum (OLF). Surgical method was categorized as posterior decompression only (PD) and circumferential decompression (CD).

Diagnosis of CSF depends on anyone of the following criteria: (1) surgical record showed intraoperative dural injury and CSF. (2) postoperative wound drainage fluid was light, and the volume was large. (3) subcutaneous puncture from the wound was clear liquid.

### Statistical analysis

Data were analyzed using Statistical Product and Service Solutions software (version 17; SPSS, Chicago, IL). Continuous variables were recorded as mean ± standard deviation, and categorical variables were expressed as frequency or percentages. An independent t test was used to analyze the difference of continuous variables. An *χ*^2^ analysis and Fisher’s exact test were used to examine the differences among categorical variables. The statistical significance was set at *p* < 0.05.

## Results

Twenty-one patients (incidence of 1.7 %) presented unplanned hospital readmission within a 90-day period and enrolled as the study group. 63 non-readmission patients were randomly selected as the control group.

**Fig. 1:**
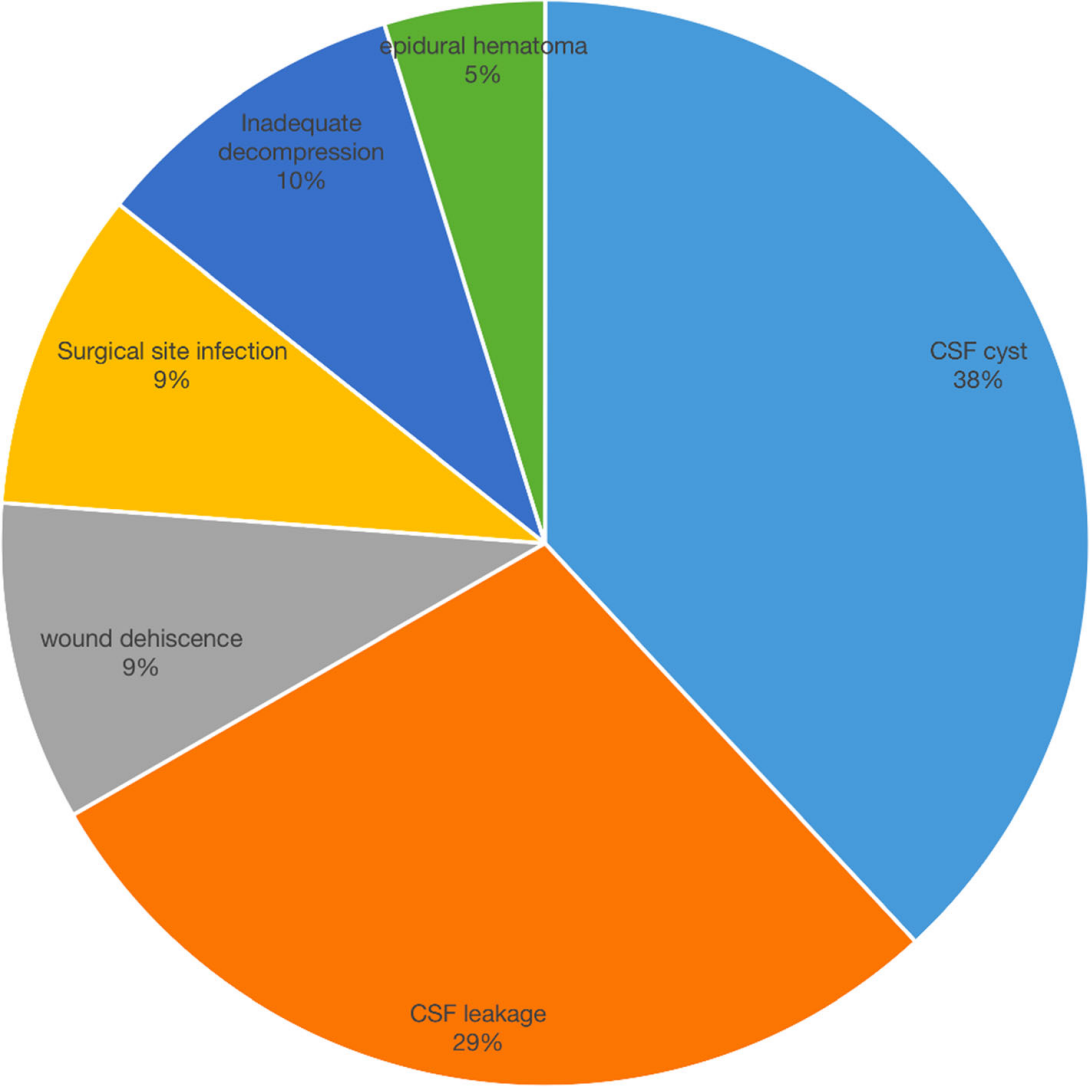
Proportion of causes of 90-day unplanned readmission after surgical treatment for TSS

**Fig. 2:**
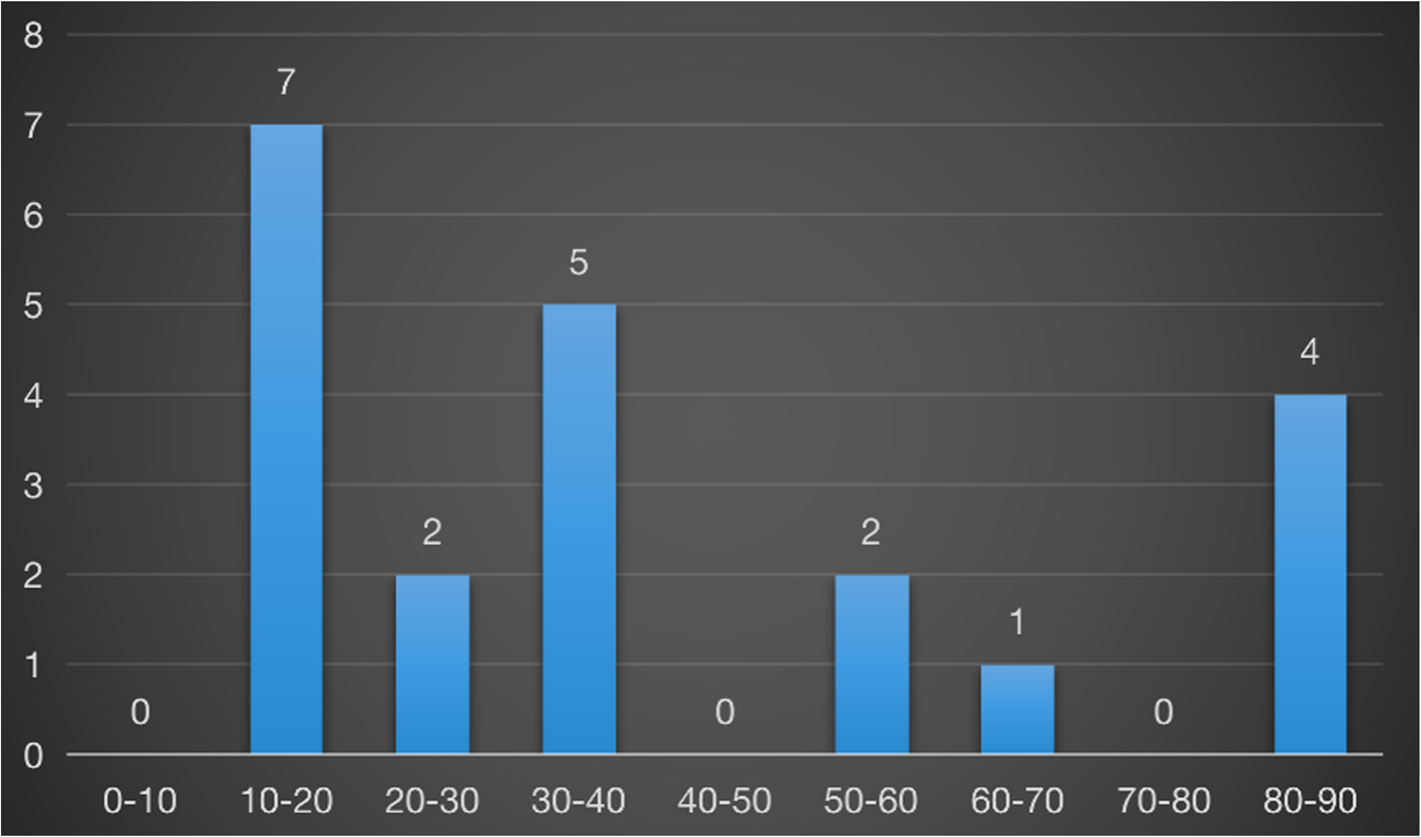
Number of patients with different duration from re-admission to the first surgery

Figure 1 shows the causes of the 90-day unplanned readmission after surgical treatment for TSS, including pseudomeningocele (8 patients; 38 %), CSF leakage combined with poor incision healing (6 patients; 29 %), wound dehiscence (2 patient; 9 %), SSI (2 patients; 9 %), spinal epidural hematoma (1 patient; 5 %), unsatisfactory symptom improvement with inadequate original surgical decompression detected by CT examination (2 patients; 9 %). Mean duration from re-admission to the first surgery was 39.6 ± 28.2 days, most of the patients readmitted at the first 40 days (66.7 %, 14/21 patients). (see Fig. 2)

**Fig. 3:**
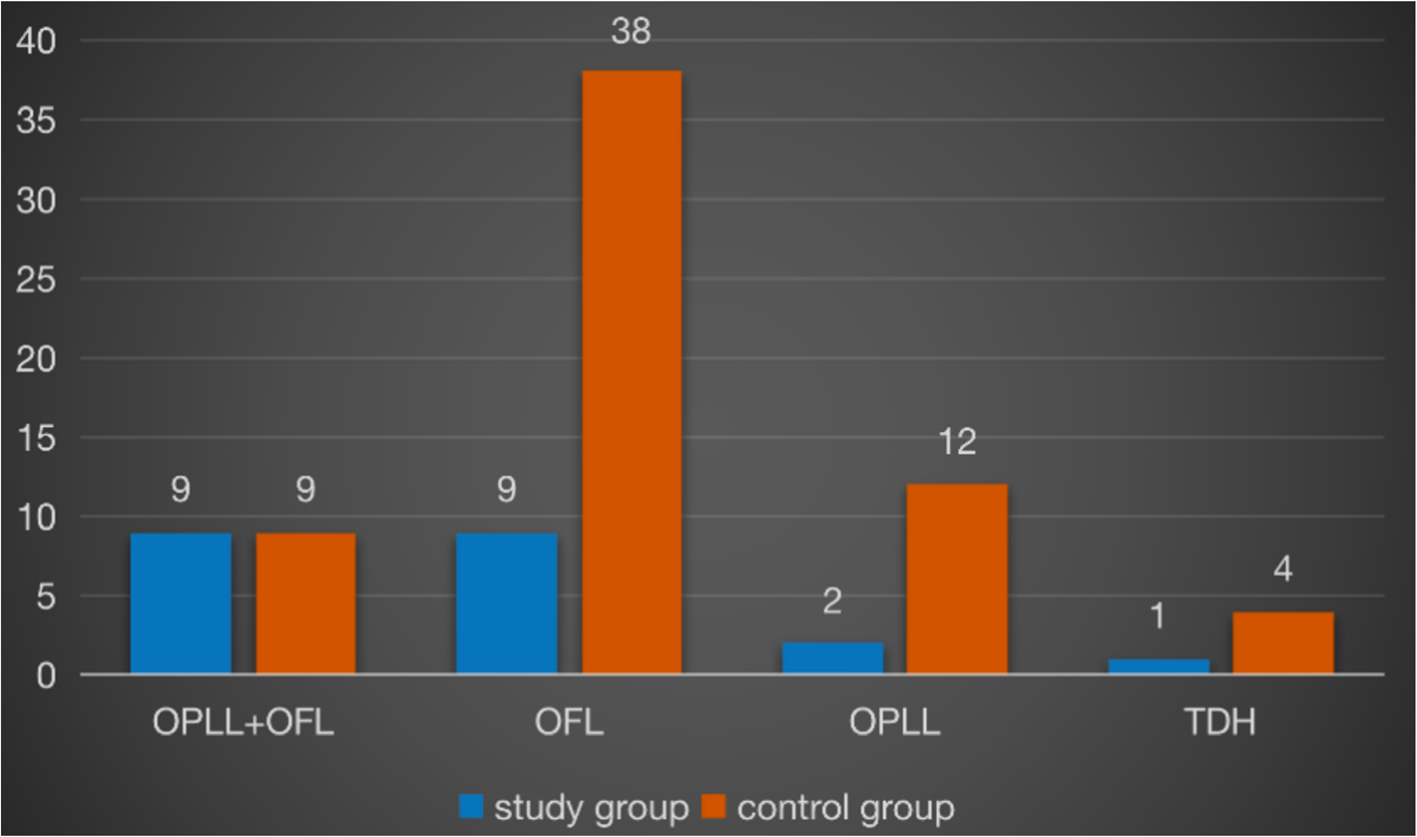
Comparison of diagnosis for the first surgery between the two groups

There were 10 males and 11 females in the study group, with mean age of 53.5 ± 9.6 years, the mean BMI was 27.6 ± 2.7 kg/m2. Diagnosis of first admission was OFL in 9 patients, OPLL + OFL in 9 patients, OPLL in 2 patients, TDH in 1 patient. Seven patients presented 3 levels thoracic spinal cord compression or less, 14 patients presented more than 3 levels thoracic spinal cord compression. Comorbidity that included hypertension, diabetes, cardiovascular disease was detected in 9 patients. There was no significant difference in age, gender, BMI, number of levels involved, comorbidity between study group and control group, except diagnosis. (Table 1) Diagnosis of OPLL + OFL was more to be seen in study group than that in control group (42.9 % vs. 14.3 %). (see Table 1; Fig. 3)

**Table 1:** Comparison of patient characteristics in the first admission between the two groups

	Study group	Control group	Statistics	*p*
Age (years)	53.5 ± 9.6	52.6 ± 8.9	0.389	0.699
Sex (Male/Female)	10/11	30/33	-	-
BMI (kg/m^2^)	27.6 ± 2.7	28.0 ± 4.0	-0.436	0.664
Diagnosis				
OPLL + OFL	9 (42.9 %)	9 (14.3 %)		
OFL	9 (42.9 %)	38 (60.3 %)		
OPLL	2 (9.5 %)	12 (19.0 %)		
TDH	1 (4.7 %)	4 (6.3 %)	4.317	0.038
Number of levels involved				
Less than 3 levels	7	25		
3 or more levels	14	38	0.269	0.604
Comorbidity				
Hypertension	4	11		
Diabetes	3	6		
Cardiovascular disease	1	2		
Total	9	19	1.143	0.285

**Fig. 4:**
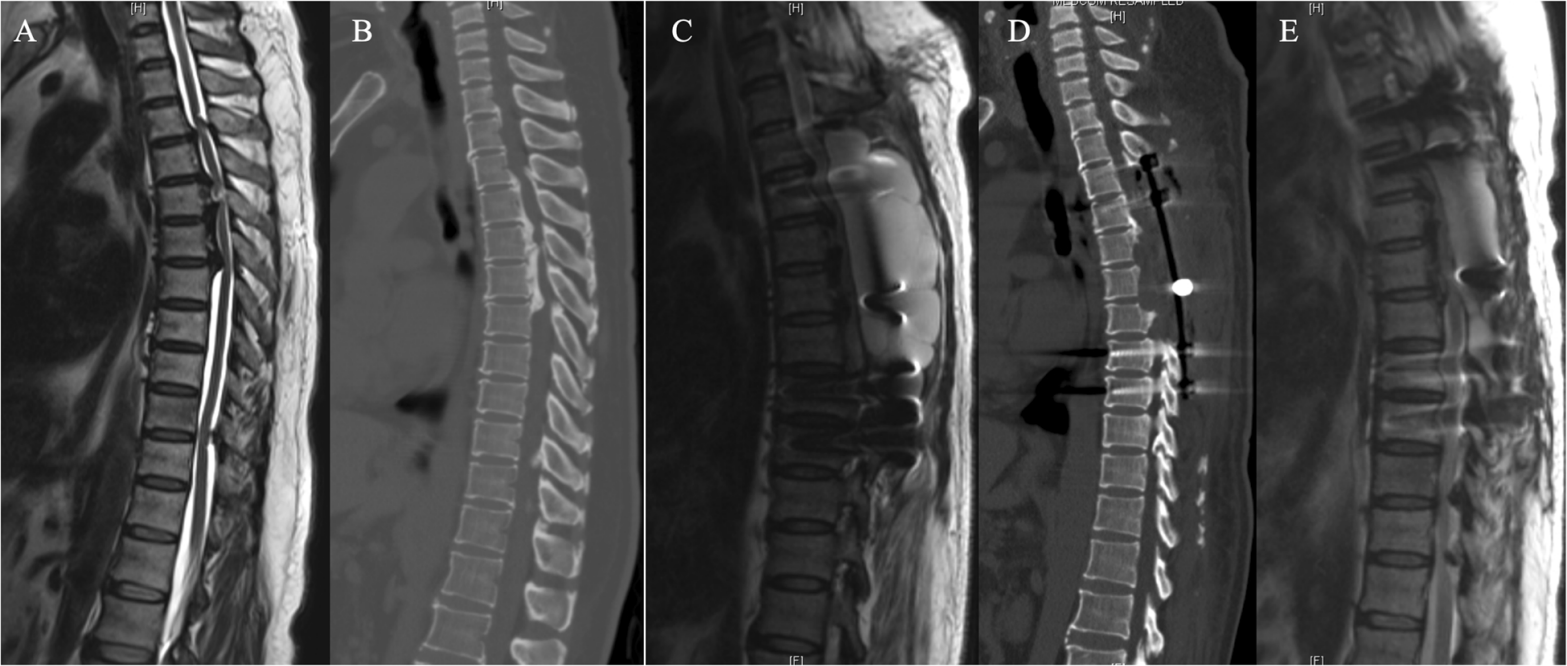
**a**, **b** Preoperative MRI and CT reveal that T4-8 OPLL and spinal cord compression. He received circumferential decompression from T4-8, with instrumentation from T3 to T9, intraoperative dural injury occurred, tight suturing of the muscle and fascia layers were implemented. Postoperatively, CSF was detected, and the wound drain was removed at 4th day. He discharged at 5th postoperative day. **c**, **d** Unplanned readmission occurred at 34th postoperative day due to the wound poor healing, MRI and CT reveal CSF around the decompression region. **e** He received revision surgery of tight suturing of the muscle and fascia layers, postoperative MRI revealed that the pseudomeningocele was cleared

In the study group, 11 patients underwent posterior decompression and 10 patients underwent circumferential decompression. Surgery time was 158.8 ± 33.3 min, blood loss was 946.2 ± 728.7 ml, extubation time was 4.6 ± 1.2 days. Intraoperative dural injury occurred in 8 patients (Fig. 4). Length of hospital stay was 12.8 ± 5.6 days. There was no significant difference in surgery time, blood loss, extubation time between study group and control group, except surgical method, rate of dural injury, length of hospital stay. Relatively more patients underwent circumferential decompression (47.6 % vs. 22.2 %), experienced dural injury (8/13 vs. 9/54), presented long hospital stay (12.8 ± 5.6 days vs. 6.2 ± 1.5 days) in study group than that in control group. (see Table 2)

**Table 2:** Comparison of surgical characteristics in the first admission between the two groups

	Study group	Control group	Statistics	*p*
Surgical method				
Posterior decompression	11 (52.4 %)	49 (77.8 %)		
Circumferential decompression	10 (47.6 %)	14 (22.2 %)	4.978	0.026
Surgery time (min)	158.8 ± 33.3	139.8 ± 69.1	0.663	0.510
Blood loss (ml)	946.2 ± 728.7	587.9 ± 882.2	1.206	0.232
Extubation time (day)	4.6 ± 1.2	4.2 ± 1.5	0.813	0.419
Dural injury (yes/no)	8/13	9/54	5.355	0.021
Length of hospital stay (day)	12.8 ± 5.6	6.2 ± 1.5	7.801	< 0.001

## Discussion

In the current study, the 90-day unplanned readmission rate after surgical treatment for TSS is 1.7 %, the causative factors include pseudomeningocele and poor incision healing followed CSF leakage, intraspinal hematoma, wound dehiscence, surgical site infection, inadequate decompression. The peak period of readmission occurred from 10 to 40 days after surgery. When compared to the non-readmitted patients, diagnosis of OPLL + OFL, circumferential decompression, dural injury, long hospital stay are more to be seen in readmitted patients, we suppose that these four factors are not independent but interrelated. Patients with OPLL combined with OFL always need circumferential decompression, which poses high risk of dural injury and increases the hospital stay subsequently. Circumferential decompression-based surgical strategy has been proved to be effective for multilevel thoracic OPLL, which could achieve both anterior and posterior decompression of the dura sac, while its postoperative courses were quite eventful, especially for patients with close adhesion of the OPLL to dura sac [20].

**Fig. 5:**
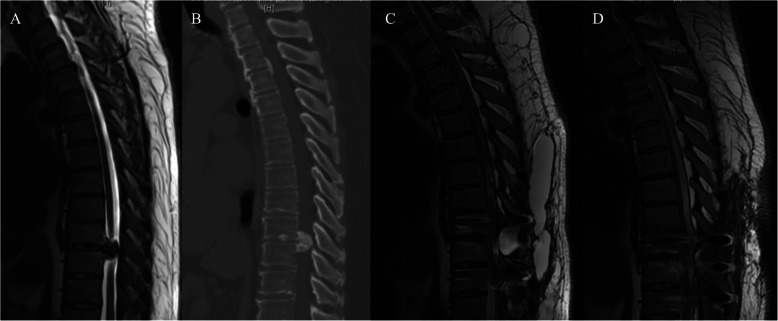
**a**, **b** Preoperative MRI and CT reveal that T10-11 OPLL and spinal cord compression. He received circumferential decompression of T10-11, with instrumentation from T10 to T12. He discharged at 4th postoperative day. Unplanned readmission occurred at 17th postoperative day due to local bulge under the incision, **c** MRI reveal CSF around the decompression region and subcutaneous pseudomeningocele. **d** He received revision surgery of tight suturing of the muscle and fascia layers, postoperative MRI revealed that the pseudomeningocele was cleared

It is unavoidable to injure the dura sac in patients with OLF and OPLL due to the adhesions between ossified ligaments and the dura sac. Moreover, ossification of dura sac could increase the technical difficulty of surgical decompression and the risk of postoperative CSF leakage [21]. Previous studies demonstrated that CSF leakage did not affect the long-term clinical outcomes, but we find that cerebrospinal fluid cyst and poor incision healing followed CSF leakage could increase the risk of unplanned hospital readmission after surgical treatment of TSS in the short term (within 90 days of the first surgery) [22, 23]. The main complaint of patients with cerebrospinal fluid cyst is local bulge under the incision (Fig. 5), headache and lower extremities weakness, and they admitted from the first month to third months after surgery, while patients with poor incision healing followed CSF leakage always readmitted within first month after surgery. For reducing the incidence of postoperative CSF leakage and the rate of unplanned admission, we suggest four possible surgical strategies. Firstly, for patients that are at high risk of dural tear in the preoperative evaluation, such as dura ossification, floating method should be recommended as the primary surgical method, instead of slitting dura procedure. Secondly, try to prevent dural tear by improving surgical instrumentation, it has been reported that the decompression surgery could be completed safely and quickly when use of ultrasonic bone curette in posterior thoracic decompression [24]. Thirdly, try to repair dural tear directly if possible using fibrin glue, gelfoam and artificial dura, and anaesthetists should ask the patient to perform a few Valsalva manoeuvres to confirm absence of CSF leakage. Fourthly, it is important to ensure tight suturing of the muscle and fascia layers [25]. Postoperatively, bedrest and compressive dressing after the removal of drainage are also necessary during the hospitalization [26].

Postoperative spinal epidural hematoma (PSEH) is a rare complication after spinal surgery with the incidence ranged from 0.1–0.4 %, often results from incomplete intraoperative haemostasis, blocked drainage tubes or coagulopathy, it can lead to devastating neurological deficits including sensory disturbance, lower extremities weakness, and bowel dysfunction [27]. PSEH often develops in a few days, especially on the day of surgery. However, on rare occasions, PSEH can occur more than three days, up to two weeks after the initial surgery. Uribe et al. defined it as the delayed PSEH, and reported the incidence was 0.17 % [28]. In the current study, a female patient experienced circumferential decompression for T3-4 OPLL combined with OFL, she discharged on postoperative day 5, while complained lower extremities weakness on day 7, then readmitted and diagnosed as delayed PSEH. We cannot make any conclusion based on only one patient data, but it should be clear that any attempt to prevent PSEH may potentially decrease the incidence of unplanned admission in surgically treated patients with TSS.

Wound dehiscence is uncommon in spine surgery, but it could result in unplanned readmission if occurred. Winward et al. reported that complications of SSI and wound dehiscence were most common readmission reasons, accounting for 15.6 % of all readmission following posterior cervical fusion [29]. Chibuikem et al. also find that SSI and wound complications were the most common causes of readmission after surgical treatment of common lumbar pathologies [5]. In the current study, only two patients presented readmission due to wound dehiscence in surgically treated patients with TSS, it is not the primary contributor, differing from the cervical and lumbar spine. Anatomically, the incision in posterior thoracic decompression surgery results in greater susceptibility to tension from paraspinal muscles due to the thoracic kyphosis, pedicle screws would further increase the tension, especially in patients with long decompression level through kyphosis apex. Consequently, there is a greater stress on the healing skin and fascial layers, finally lead to wound dehiscence. Ando et al. have proved that dekyphosis using multilevel Ponte osteotomies could provide indirect decompression of the spinal cord [30]. We suggest that thoracic kyphosis decrease may also prevent wound dehiscence by reducing the tension from paraspinal muscles and may decrease the incidence of unplanned readmission in surgically treated patients with TSS.

SSI is a relatively common complication after spinal surgery, with reported incidence ranges from 0.1 % to 10.9 % [16]. SSI increases the morbidity, mortality, length of hospital stay, readmission, and health care costs. In the current study, two patients readmitted due to SSI, demonstrating that trying to prevent SSI may decrease the rate of readmission. It has been proved that risk factors of SSI include age, ASA score, obesity, diabetes, smoking, radiation therapy, psoriasis, chronic skin conditions, use of instrumentation, bone graft harvesting [31]. The identified predictors of SSI can improve identification of high risk patients and provide a key target for intervention.

Inadequate original surgical decompression is rarely reported in the previous literature, there is a paucity of published data on the incidence of patients with myelopathy who have undergone a surgical decompression for TSS that was subsequently found to be inadequate. This lack of data may be due to the postoperative CT is not commonly obtained unless, or until, a subsequent neurologic decline is noted. Definitely, revision surgery may be required for most of the patients that with inadequate original surgical decompression, then lead the unplanned admission. In the current study, two OLF patients experienced unsatisfactory symptom relief and functional improvement at three months follow up, then confirmed inadequate original surgical decompression through CT. We suppose this type of readmission should be classified as iatrogenic, because it is possibly derived from the improper surgical plan by surgeons, more care should be taken in the preoperative surgical planning, one more segment decompression both at caudal and cephalad could decrease the risk of inadequate original surgical decompression.

This retrospective study has several limitations. First, risk factors for readmission were not assessed through Logistic regression analysis because of the relatively small sample size. Second, this is a single institution study, the conclusion may be not necessarily generalizable or representative of the population at large, but it could provide the advantage of consistency in surgical practice and clinical care.

## Conclusions

The incidence of 90-day unplanned readmission after surgical treatment for TSS is 1.7 %, CSF leakage and pseudomeningocele were the most common causes of readmission, the peak period of readmission occurred from 10 to 40 days after surgery, patients should be closely followed up within this period.

## Data Availability

The datasets will be available from the corresponding author if required and being granted permission from Ethics Committee of the Peking University Third Hospital.

## Abbreviations

TSS: Thoracic spinal stenosis
SSI: Surgical site infections
CSF: Cerebrospinal fluid
BMI: Body mass index
TDH: Thoracic disc herniation
OPLL: Ossification of posterior longitudinal ligament
OLF: Ossification of thoracic ligamentum flavum
PD: Posterior decompression
CD: Circumferential decompression
PSEH: Postoperative spinal epidural hematoma
